# Supplementary material for: Novel Multitarget Hydroxamic Acids with a Natural Origin CAP Group against Alzheimer’s Disease: Synthesis, Docking and Biological Evaluation
Source: Pharmaceutics. 2021 Nov 8;13(11):1893. doi: 10.3390/pharmaceutics13111893 (PMC8623418; doi:10.3390/pharmaceutics13111893)
Supplement: Supplementary file 1 [file pharmaceutics-13-01893-s001.zip › pharmaceutics-1437532-supplementary.pdf]

pharmaceutics

Supplementary Materials: Novel Multitarget Hydroxamic Acids with a Natural Origin CAP Group against Alzheimer's Disease: Synthesis, Docking and Biological Evaluation

Margarita Neganova , Yulia Aleksandrova , Evgenii Suslov , Evgenii Mozhaitsev , Aldar Munkuev , Dmitry Tsypyshev , Maria Chicheva , Artem Rogachev , Olga Sukocheva , Konstantin Volcho and Sergey Klochkov

Molecular docking

RCSB Protein Data Bank codes

A crystal structure of HDAC6 domain 2 in complex with reference ligand trichostatin A was extracted from RSCD PDB [https://www.rcsb.org], (ID: 5EDU [Hai, Y., & Christianson, D. W. (2016). Histone deacetylase 6 structure and molecular basis of catalysis and inhibition. Nature Chemical Biology, 12(9), 741–747. doi:10.1038/nchembio.2134]).

Ligand preparation

Molecular docking of compounds **2**, **11-16**, **23-25** into the 5EDU binding site of HDAC6 domain 2 was performed using the Schrödinger Suites 2018-1 program complex (Schrödinger Release 2018-1: Maestro, Schrödinger, LLC, New York, NY, 2018). All structures were sketched by means the 'Maestro' molecular editor and prepared for subsequent procedures via 'LigPrep' module: geometries were optimized using OPLS3e force field [Harder, E.; Damm, W.; Maple, J.; Wu, C.; Reboul, M.; Xiang, J.Y.; Wang, L.; Lupyan, D.; Dahlgren, M.K.; Knight, J.L.; Kaus, J.W.; Cerutti, D.; Krilov, G.; Jorgensen, W.L.; Abel, R.; and Friesner, R.A. OPLS3: a force field providing broad coverage of drug-like small molecules and proteins // J. Chem. Theory Comput. 12, 281-296 (2016)], and their ionization states were generated at pH 7.0 ± 2.0 using 'Epik' [Greenwood, J. R.; Calkins, D.; Sullivan, A. P.; Shelley, J. C. Towards the comprehensive, rapid, and accurate prediction of the favorable tautomeric states of drug-like molecules in aqueous solution // J. Comput. Aided Mol. Des. 24, 591-604 (2010); Shelley, J.C.; Cholleti, A.; Frye, L; Greenwood, J.R.; Timlin, M.R.; Uchimaya, M. Epik: a software program for pKa prediction and protonation state generation for drug-like molecules // J. Comp. Aided Mol. Des. 21, 681-691 (2007)].

Protein preparation

Following additional procedures were carried out with the respective protein structure (PDB ID: 5EDU) using the 'Protein Preparation Wizard' module [Sastry, G.M.; Adzhigirey, M.; Day, T.; Annabhimoju, R.; Sherman, W. Protein and ligand preparation: Parameters, protocols, and influence on virtual screening enrichments // *J. Comput. Aid. Mol. Des*. 27(3), 221-234 (2013)]: unspecified side chains and loops were restored using the 'Prime' utility [Jacobson, M. P.; Pincus, D. L.; Rapp, C. S.; Day, T. J. F.; Honig, B.; Shaw, D. E.; Friesner, R. A. A Hierarchical Approach to All-Atom Protein Loop Prediction // *Proteins: Structure, Function and Bioinformatics*, 55, 351-367 (2004). Jacobson, M. P.; Friesner, R.A.; Xiang, Z.; Honig, B. On the Role of Crystal Packing Forces in Determining Protein Sidechain Conformations // *J. Mol. Biol.*, 320, 597-608 (2002)]. After these modifications, the search for hydrogen bonds was performed and hydrogen bonds were re-declared. Water and other irrelevant molecules were removed, and restrained minimization of the geometric structure of the complexes was performed. A number of receptor grids were generated to define a ligand binding site for subsequent docking analysis. A grid box of 20×20×20 Å was created for 'ligand-receptor' complex, centered on the center of mass of the ligand in the selected crystal structure covering the 5EDU binding site. A scaling factor of 1.0 and a partial charge threshold of 0.25 were used

during the generation of the grid boxes. Also, all possible hydroxyl and thiol groups in the vicinity of the active center of the receptor that could undergo rotation were marked as rotatable.

Docking protocols and calculations

The generated grid boxes were then used for re-docking of reference ligand and docking of set of compounds using the Glide extra-precision protocol at the first step [Friesner, R.A.; Murphy, R.B.; Repasky, M.P.; Frye, L.L.; Greenwood, J.R.; Halgren, T.A.; Sanschagrin, P.C.; Mainz, D.T. Extra Precision Glide: Docking and Scoring Incorporating a Model of Hydrophobic Enclosure for Protein-Ligand Complexes // J. Med. Chem., 49, 6177–6196 (2006); Halgren, T. A.; Murphy, R. B.; Friesner, R. A.; Beard, H. S.; Frye, L. L.; Pollard, W. T.; Banks, J. L. Glide: A New Approach for Rapid, Accurate Docking and Scoring. 2. Enrichment Factors in Database Screening // J. Med. Chem., 47, 1750–1759 (2004); Friesner, R. A.; Banks, J. L.; Murphy, R. B.; Halgren, T. A.; Klicic, J. J.; Mainz, D. T.; Repasky, M. P.; Knoll, E. H.; Shaw, D. E.; Shelley, M.; Perry, J. K.; Francis, P.; Shenkin, P. S. Glide: A New Approach for Rapid, Accurate Docking and Scoring. 1. Method and Assessment of Docking Accuracy // J. Med. Chem., 47, 1739–1749 (2004)]. The best poses obtained as results of this procedure then were used for following MM_GBSA calculations, in order to obtain the ΔG_{bind} values. To calculate the binding energy between ligand and the Sigma1R in the 'ligand-protein complex' (solvent - water, the flexibility of the protein is limited within a radius of 5 Å from the ligand), the generalized Born model (GB) was used, taking into account the available surface area (SA) in the context of molecular mechanics (MM): ΔGbind (MM_GBSA).

Ki calculation

The values of inhibition constant Ki were calculated according to the formula:

$$K_i = e^{-\frac{\Delta G_{bind}}{RT}}, \text{ where}$$

$$\Delta G_{bind} \text{ - } (cal/mol); \text{ T} = 298\ K; \text{ R} = 1.987\ \frac{cal}{mol \times K}.$$

Table S1. Results of docking of the synthesized compounds **2, 11-16, 23-25** into the zinc-containing HDAC6 binding site (5EDU); RMSD of reference Trichostatin A is 1.5030 Å.

Compd's ID*	MM_GBSA ΔG_{bind}, kcal/mol	H-bonds	Other interactions	Hydrophobic interactions
Trichostatine	-64.34	GLY619		PRO501 PRO608 PHE620 PHE680 LEU749 TYR782
24	-64.05	HIS610 TYR782	π-π: PHE620	PRO501 PHE620 PHE680 LEU749 TYR782
11	-58.90	HIS610 HIS651 TYR782	---	PRO608 PHE620 PHE679 PHE680 LEU749 TYR782
15	-57.99	HIS610 HIS651 TYR782	---	PHE620 PHE679 PHE680 LEU749 TYR782
14	-57.38	HIS610 HIS651 TYR782	---	PRO608 PHE620 PHE679 PHE680 LEU749 TYR782
13	-53.69	HIS610 HIS651 TYR782	---	PHE620 PHE679 PHE680 LEU749 TYR782
16	-52.50	HIS610 HIS651 TYR782	---	PRO608 PHE620 PHE679 PHE680 LEU749 TYR782
12	-49.14	HIS610 HIS651 TYR782	---	PRO608 PHE620 PHE679 PHE680 LEU749 TYR782

25	-46.18	SER568 GLY619 TYR782	π-π: PHE680	PRO608 PHE620 CYS621 PHE680 TYR782
23	-45.65	GLY619	---	PRO501 PRO608 PHE620 CYS621 PHE680 LEU749 TYR782
2	-39.36	HIS610 TYR782	---	PRO608 PHE620 PHE680 LEU749 TYR782

* - in order of increasing of ΔG_{bind} values.

Table S2. Calculated pIC_{50} and pKi values.

Compd's ID	pIC_{50}	pKi
24	4.814457845	-47.0010908
11	6.161150909	-43.22192425
15	6.017728767	-42.55414919
14	5.348721986	-42.10651975
13	5.025028006	-39.39872857
16	6.13076828	-38.52548426
12	5.391473966	-36.05985327
25	5.185752404	-33.88774978
23	4.744004273	-33.49882584
2	4.0	-28.88310592

^{1}H and ^{13}C NMR spectra

^{1}H and ^{13}C NMR spectra were recorded on a Bruker AV-300 spectrometer (300.13 MHz and 75.46 MHz respectively), Bruker AV-400 (400.13 MHz and 100.61 MHz), Bruker DRX-500 (500.13 MHz and 125.76 MHz).

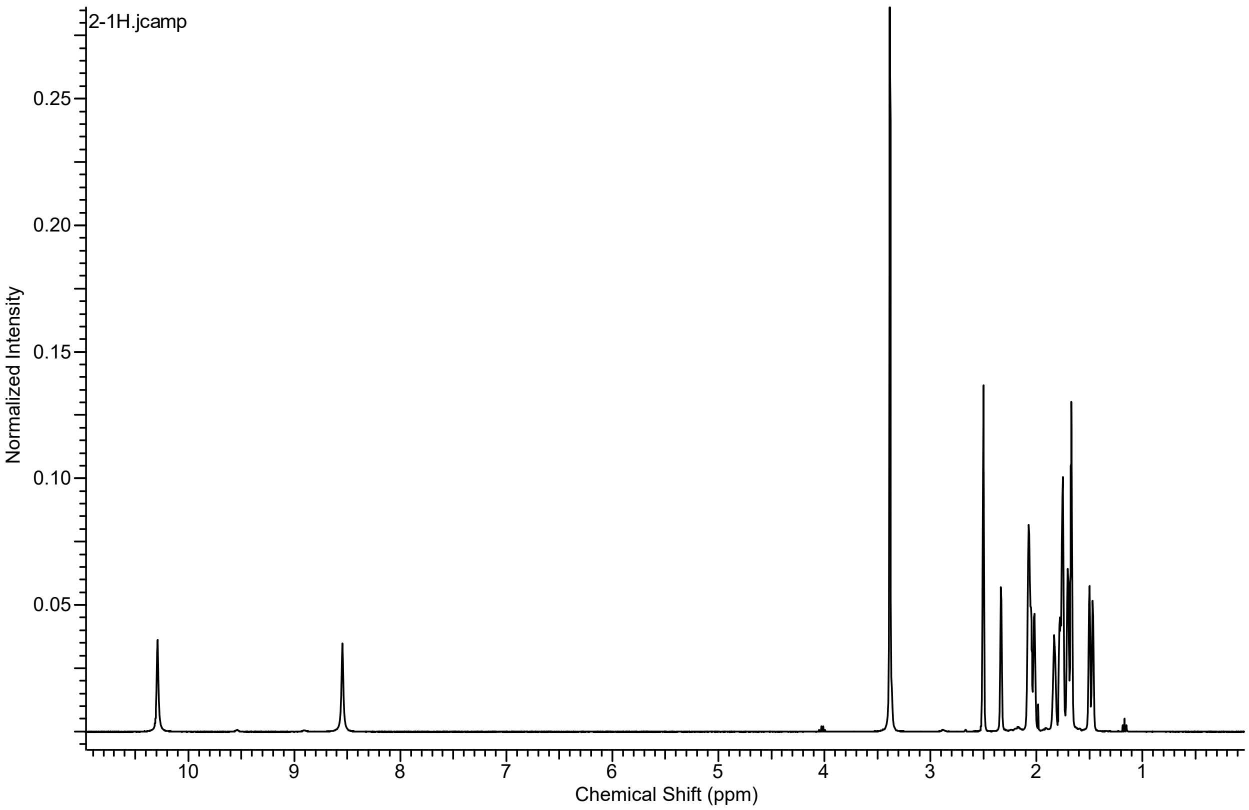

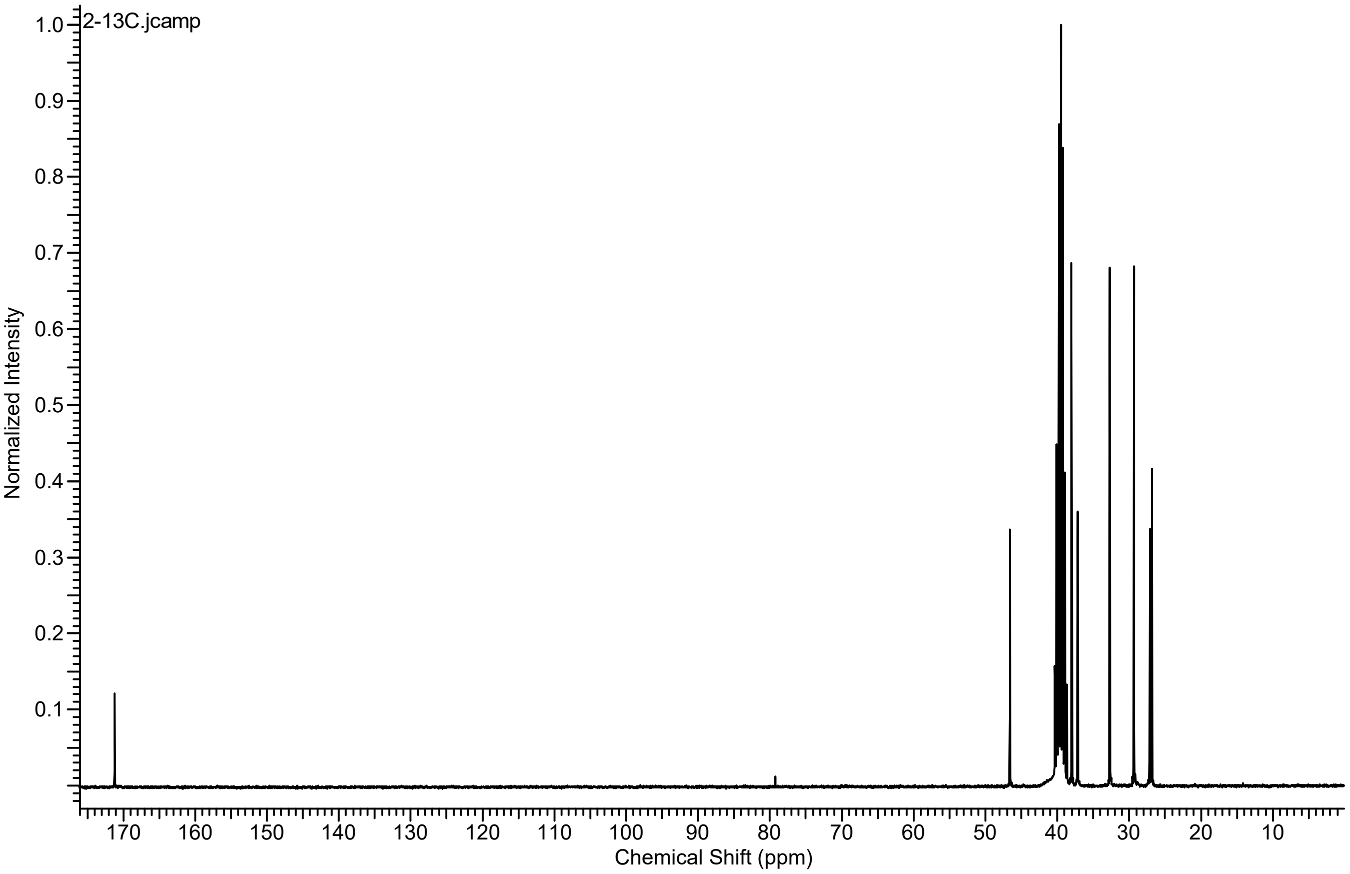

2-13C.jcamp
Normalized Intensity
Chemical Shift (ppm)

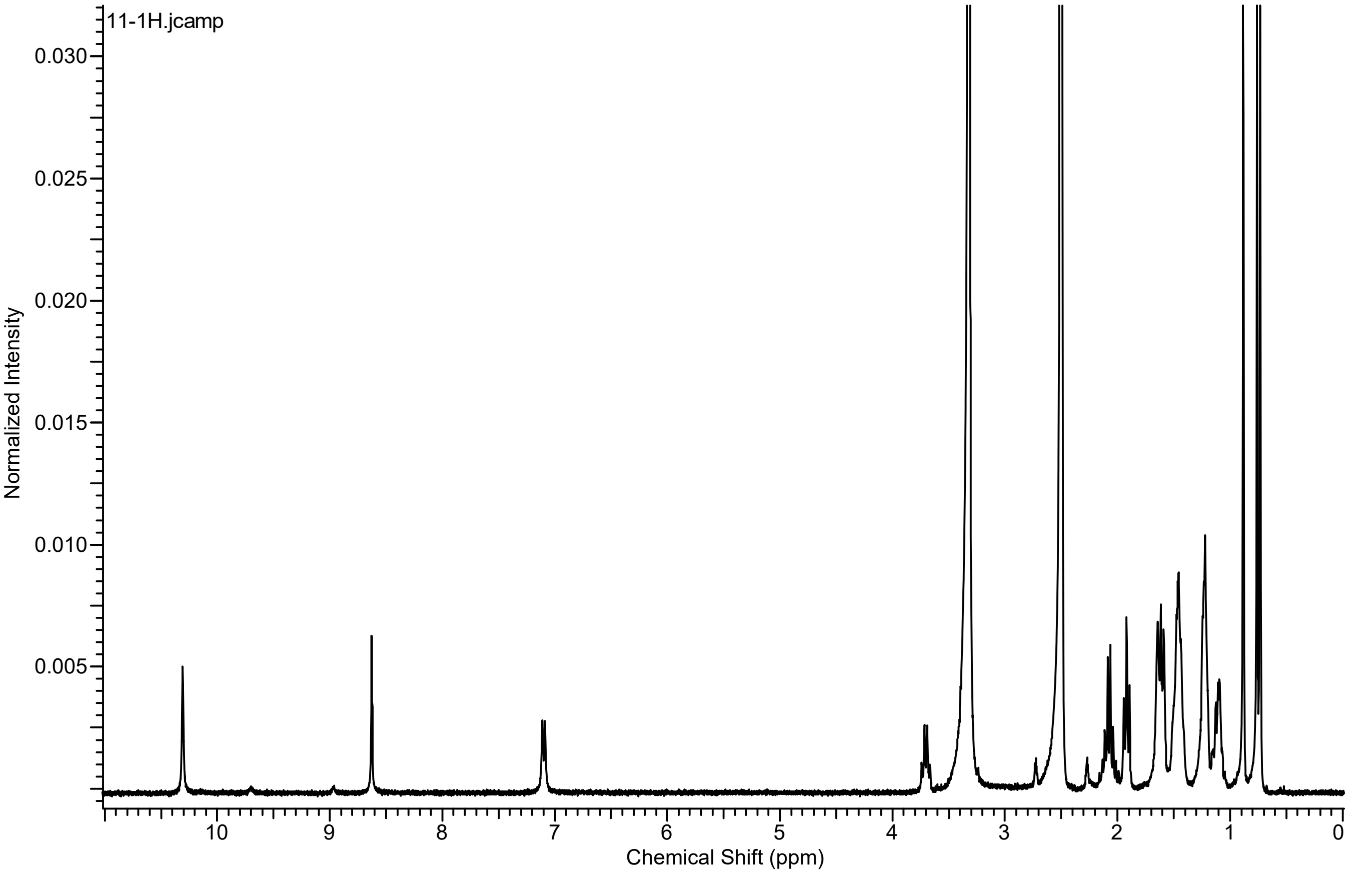

11-1H.jcamp
Normalized Intensity
Chemical Shift (ppm)

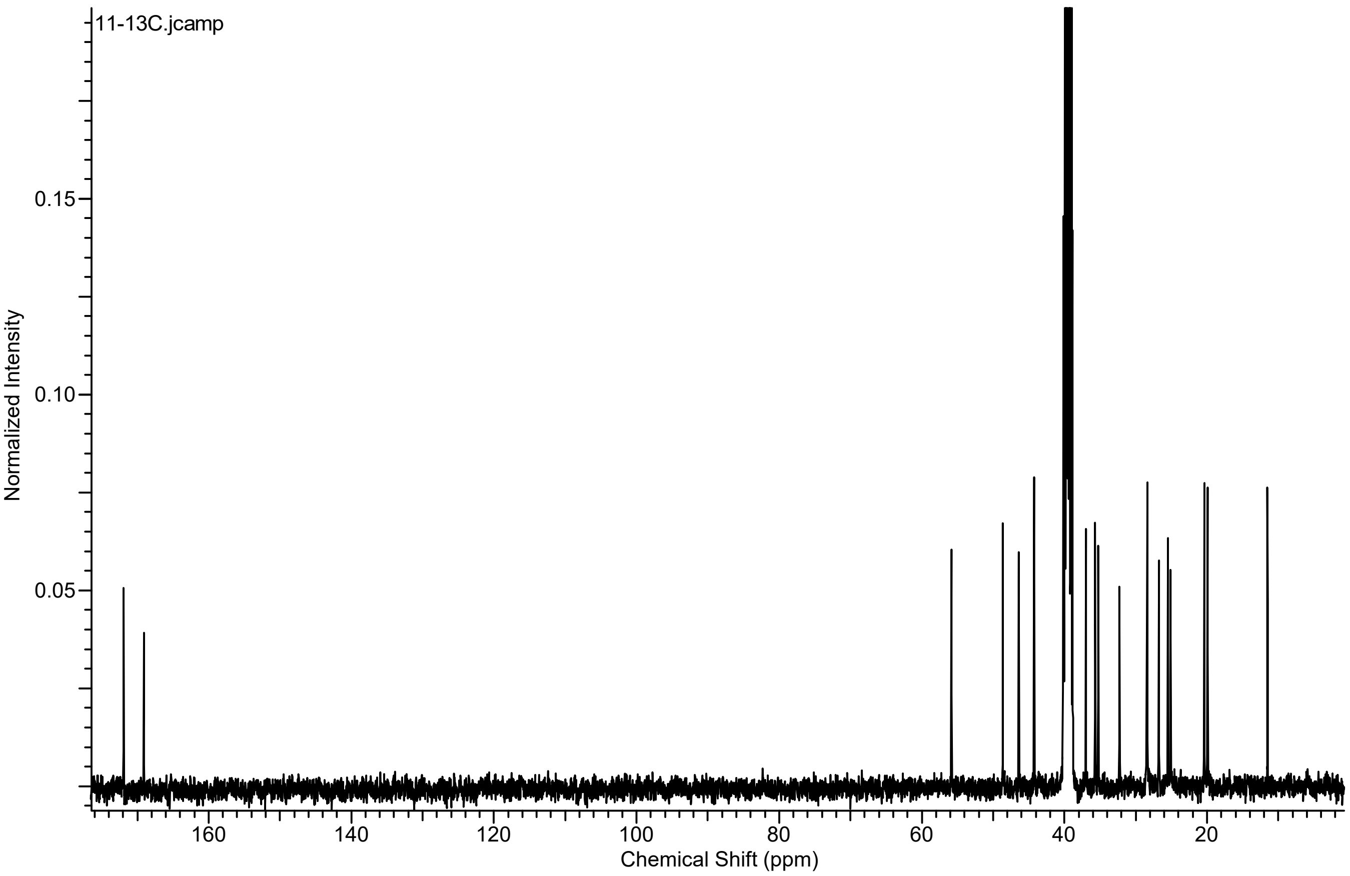
11-13C.jcamp
Normalized Intensity
0.15
0.10
0.05
160
140
120
100
80
60
40
20
Chemical Shift (ppm)

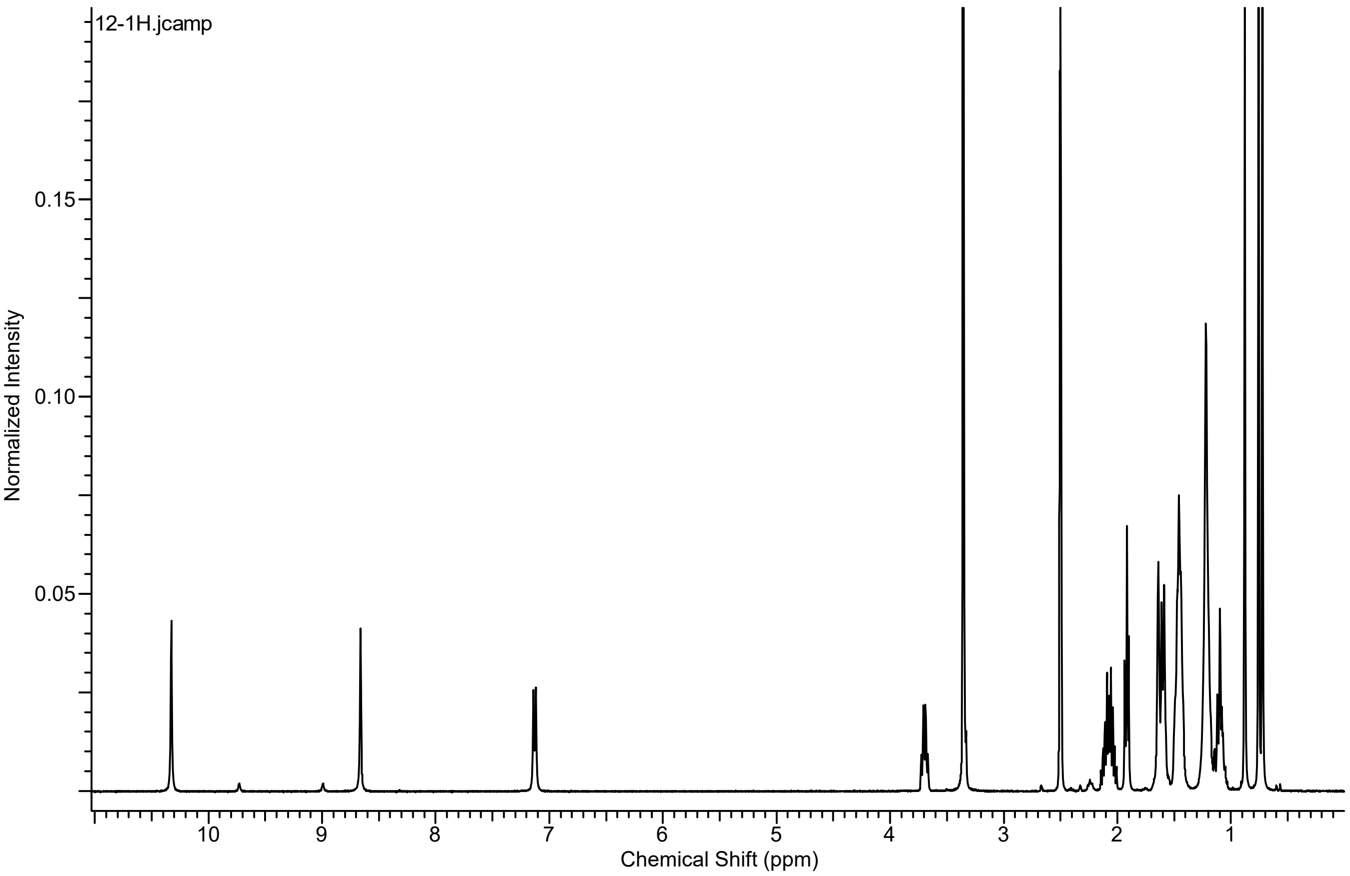
12-1H.jcamp
Normalized Intensity
0.15
0.10
0.05
10
9
8
7
6
5
4
3
2
1
Chemical Shift (ppm)

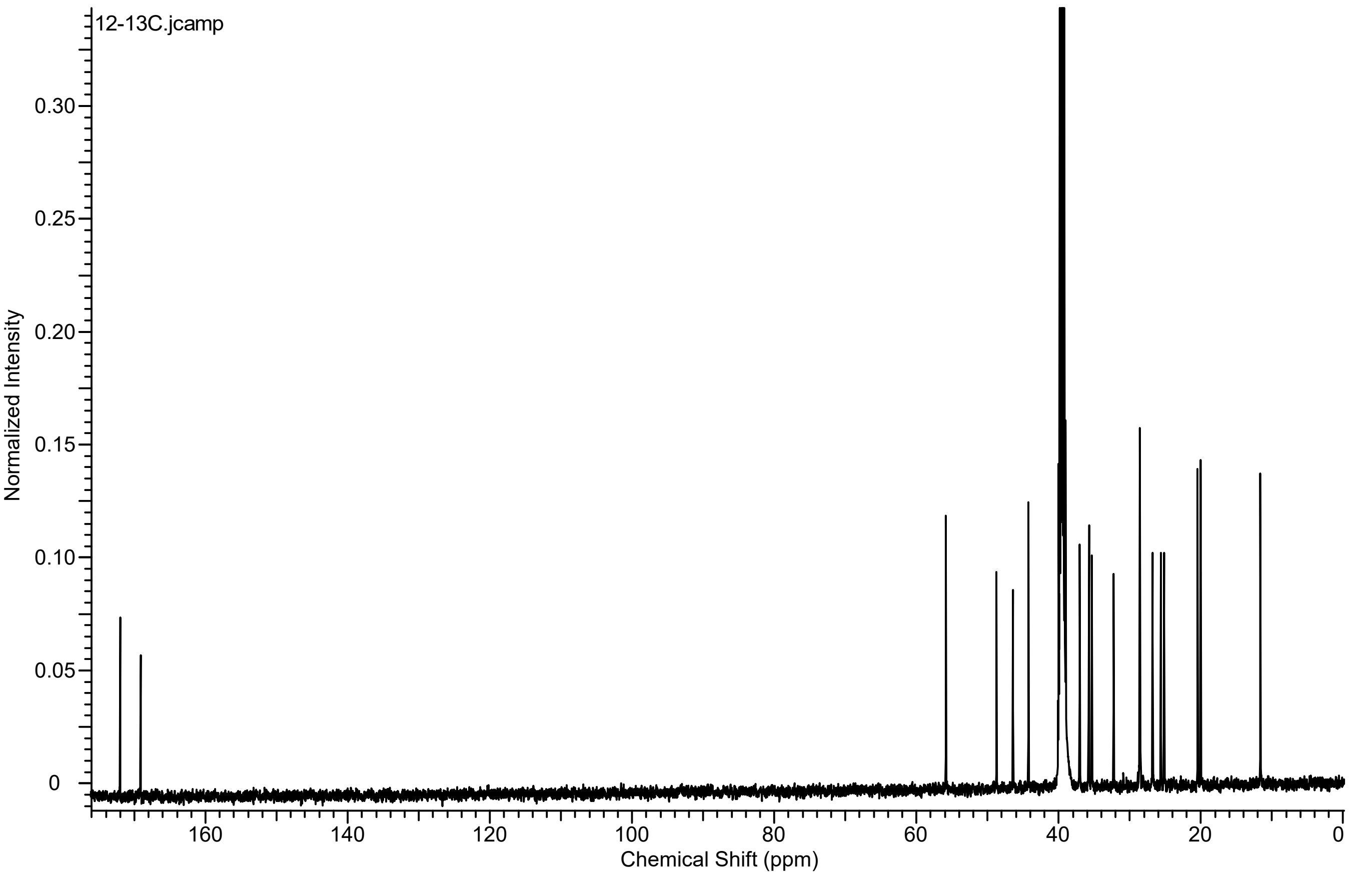
12-13C.jcamp
Normalized Intensity
0.30
0.25
0.20
0.15
0.10
0.05
0
160
140
120
100
80
60
40
20
0
Chemical Shift (ppm)

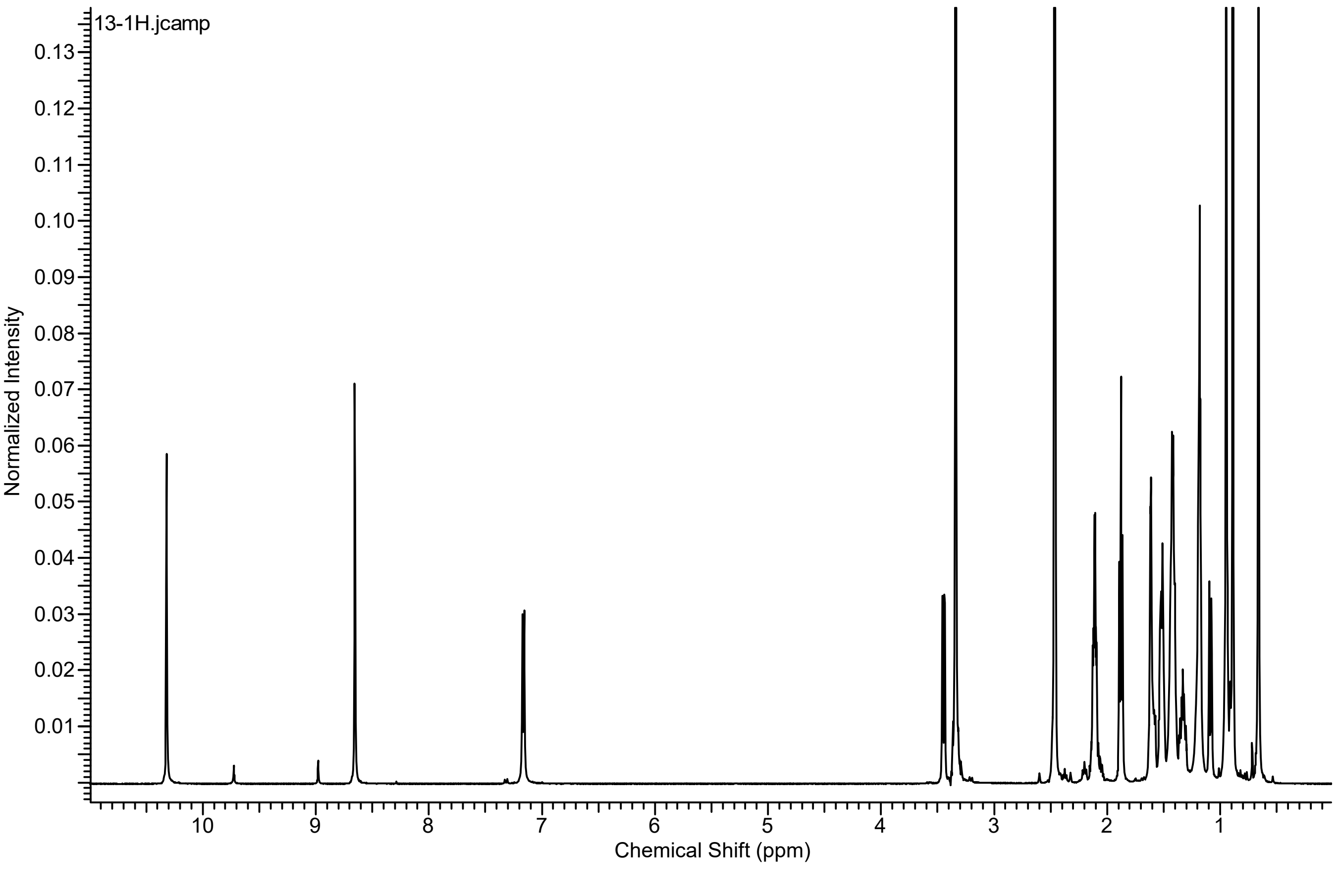
13-1H.jcamp
Normalized Intensity
0.13
0.12
0.11
0.10
0.09
0.08
0.07
0.06
0.05
0.04
0.03
0.02
0.01
10
9
8
7
6
5
4
3
2
1
Chemical Shift (ppm)

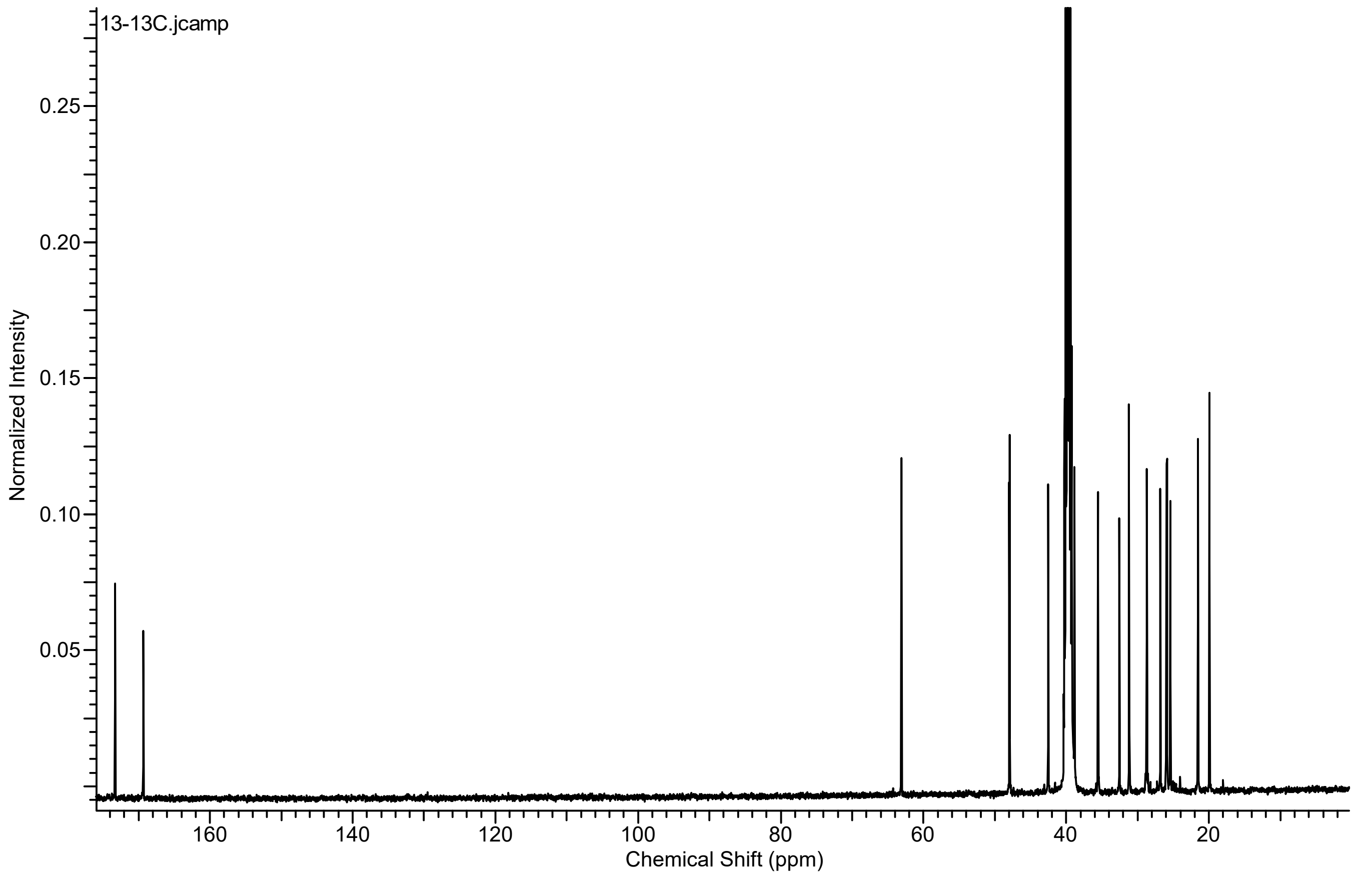
13-13C.jcamp
Normalized Intensity
0.25
0.20
0.15
0.10
0.05
160
140
120
100
80
60
40
20
Chemical Shift (ppm)

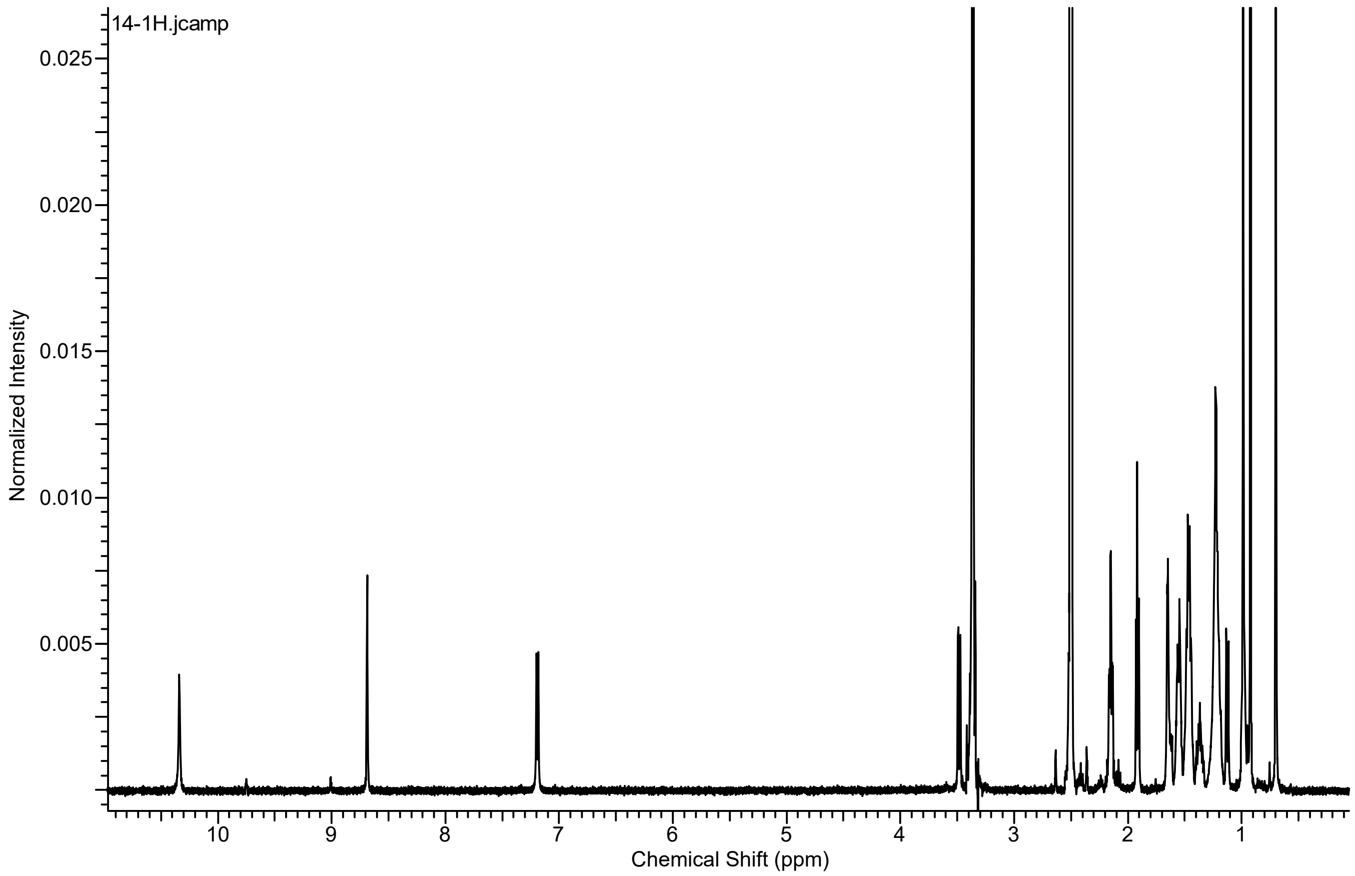
14-1H.jcamp
Normalized Intensity
0.025
0.020
0.015
0.010
0.005
10
9
8
7
6
5
4
3
2
1
Chemical Shift (ppm)

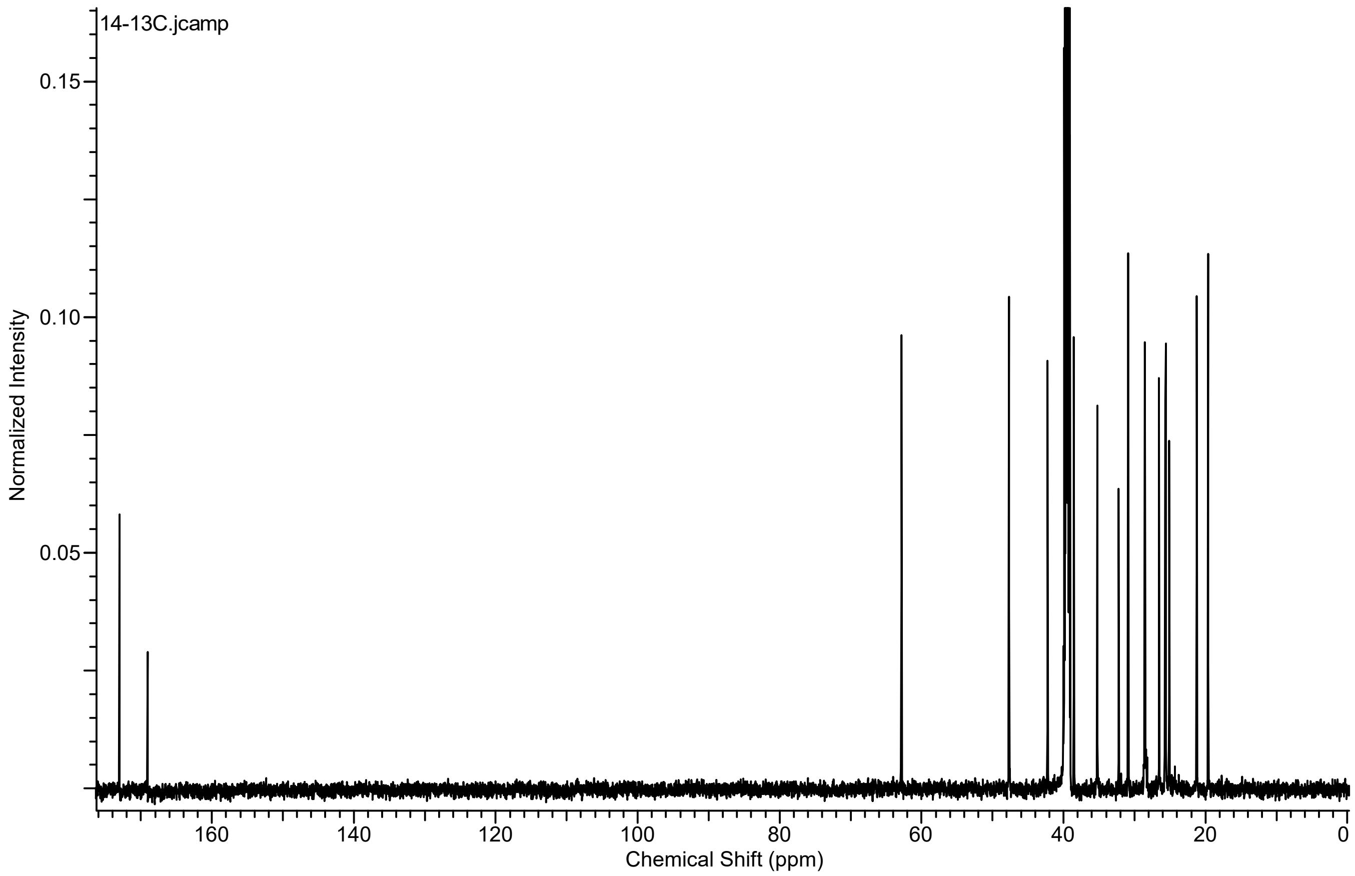
14-13C.jcamp
Normalized Intensity
0.15
0.10
0.05
160
140
120
100
80
60
40
20
0
Chemical Shift (ppm)

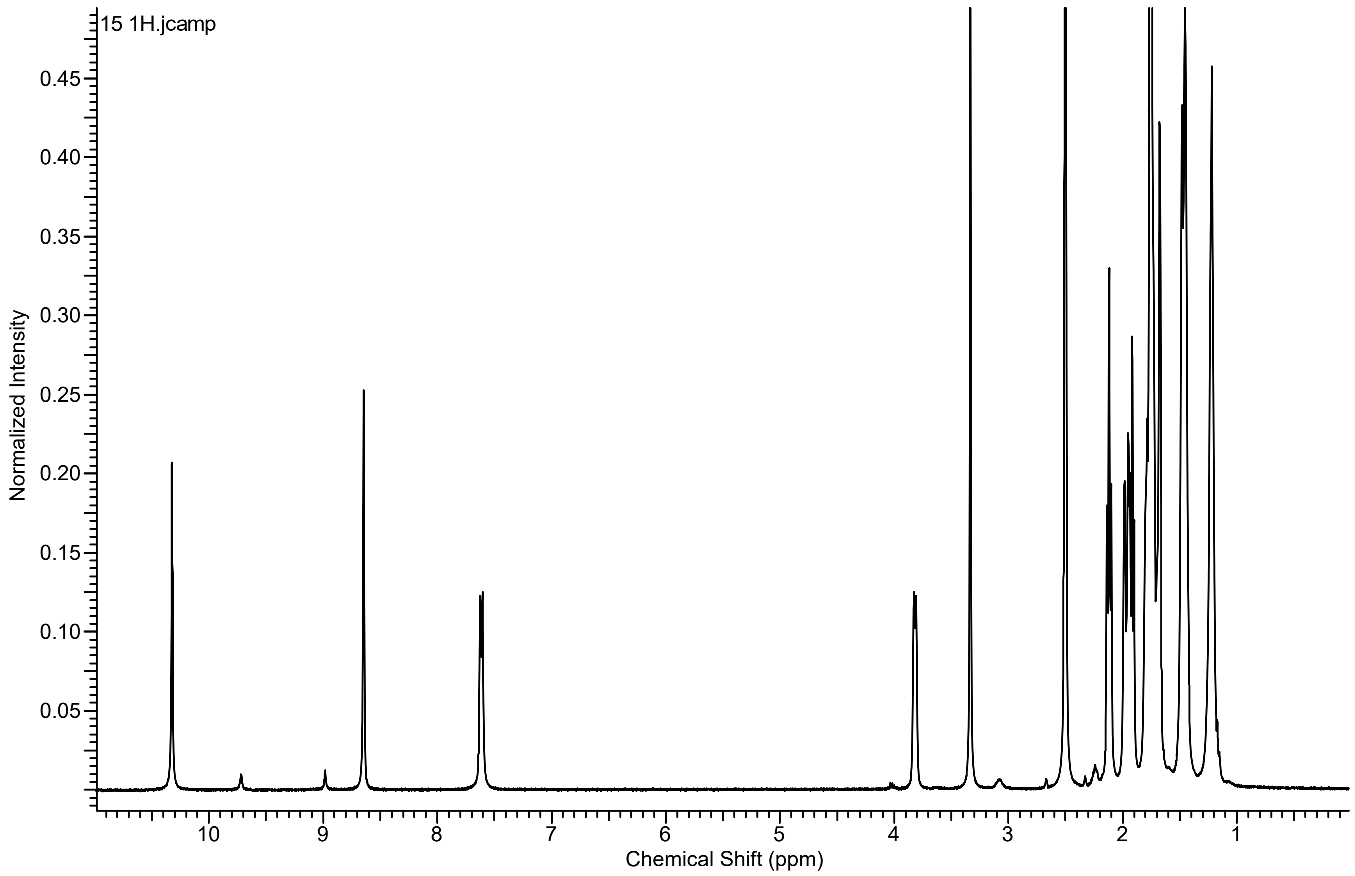
15 1H.jcamp
Normalized Intensity
0.45
0.40
0.35
0.30
0.25
0.20
0.15
0.10
0.05
10
9
8
7
6
5
4
3
2
1
Chemical Shift (ppm)

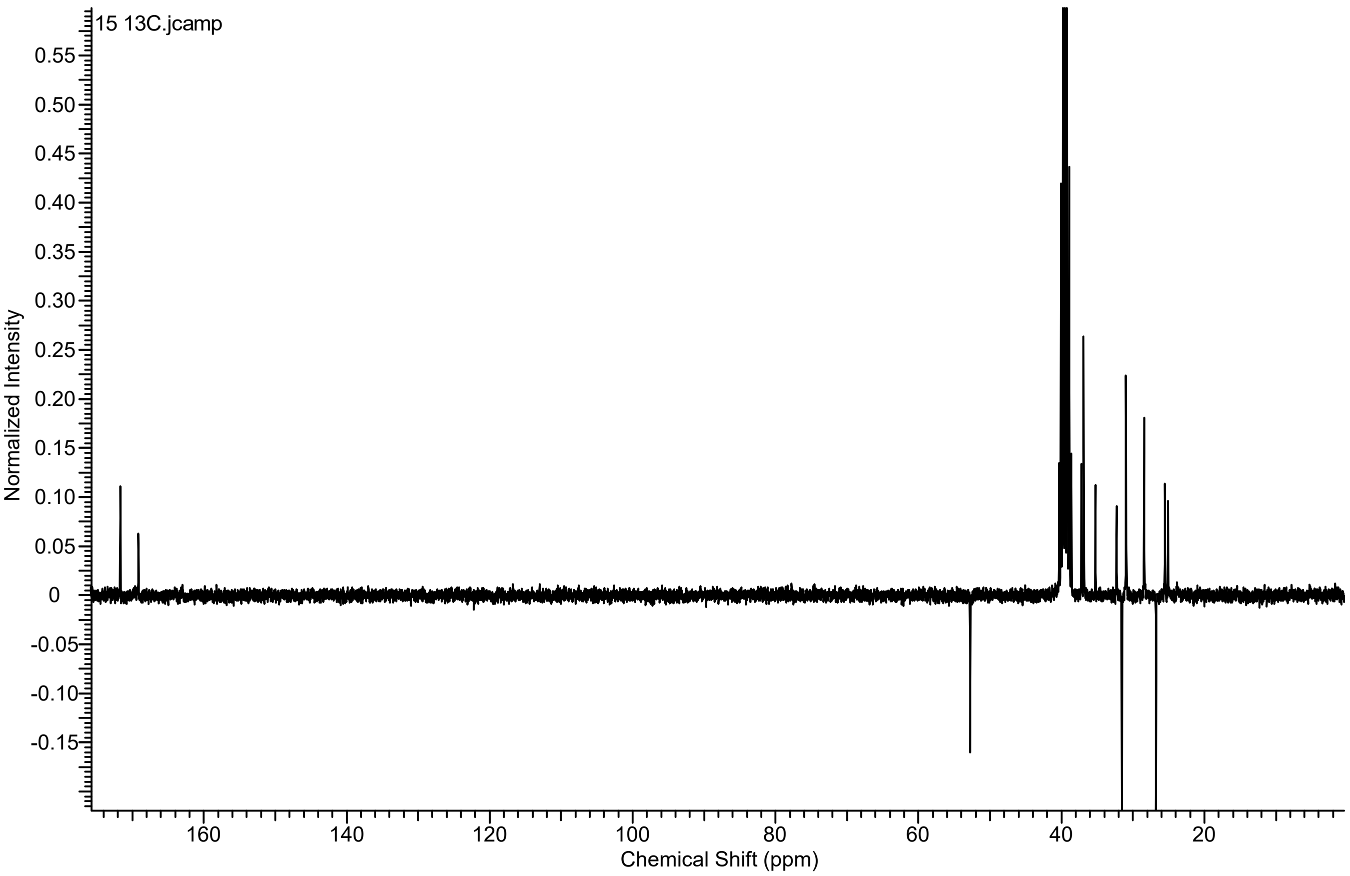
15 13C.jcamp
Normalized Intensity
Chemical Shift (ppm)

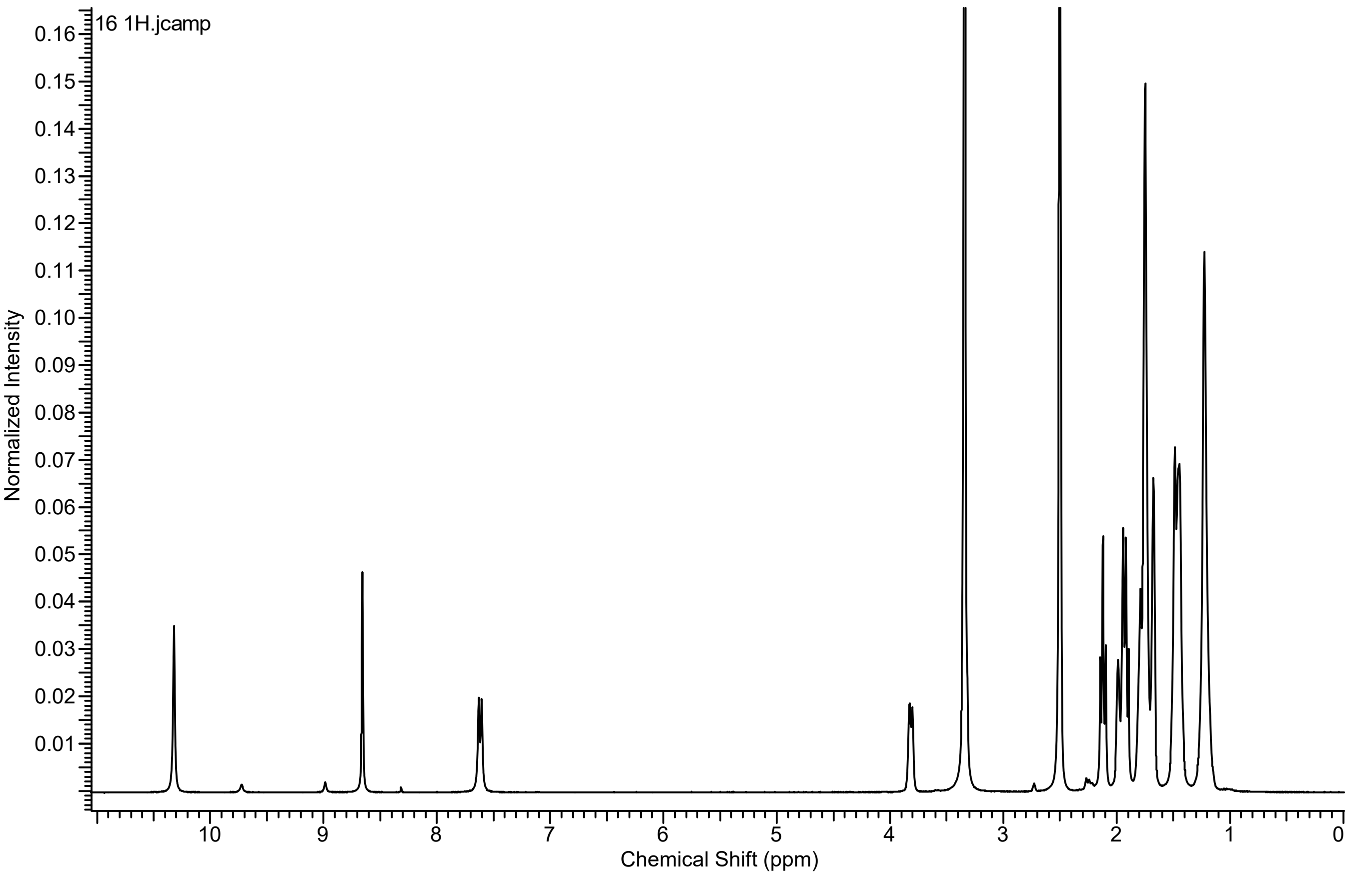
16 1H.jcamp
Normalized Intensity
Chemical Shift (ppm)

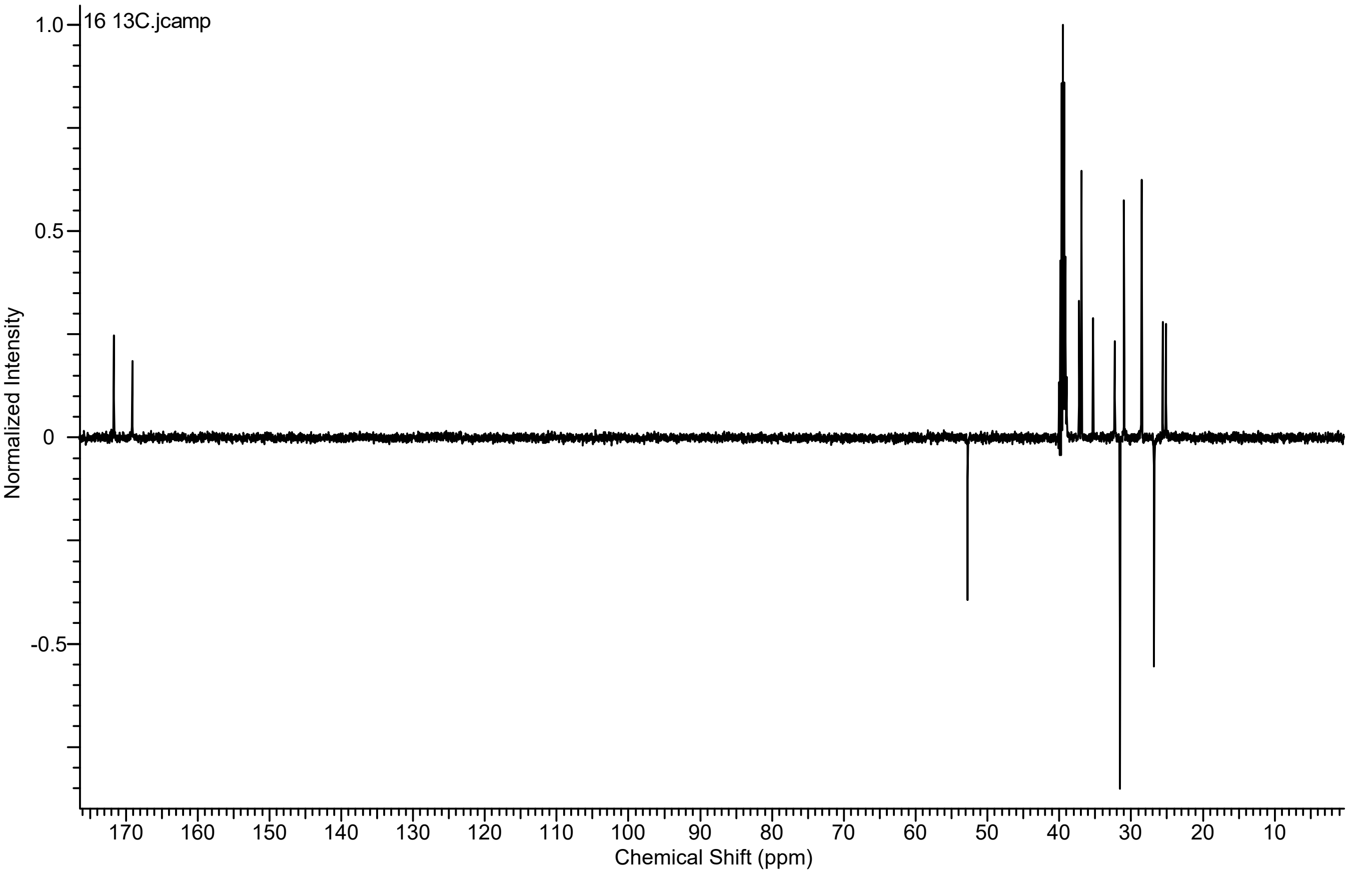
16 13C.jcamp
Normalized Intensity
Chemical Shift (ppm)

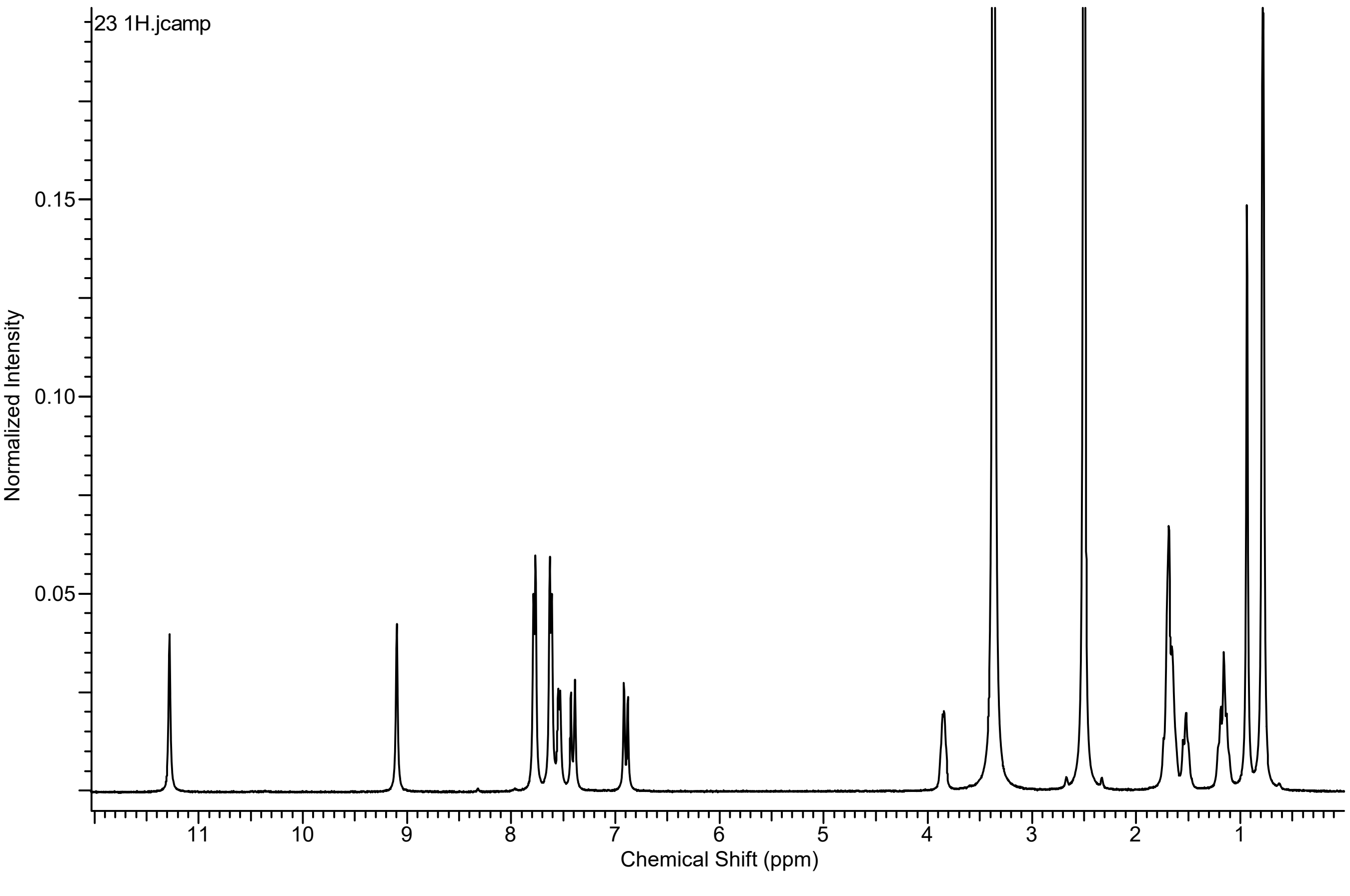
23 1H.jcamp
Normalized Intensity
Chemical Shift (ppm)

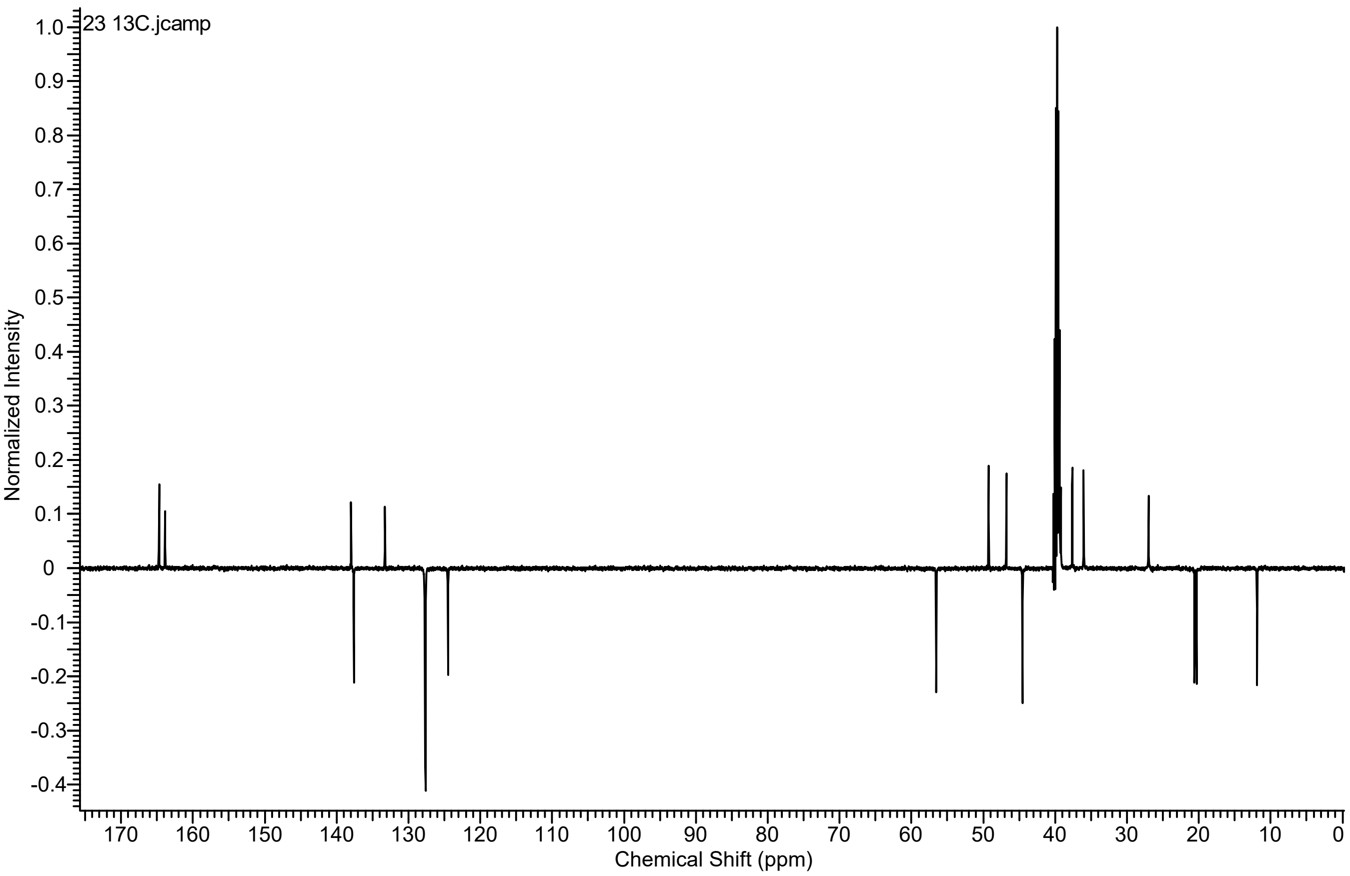
23 13C.jcamp
Normalized Intensity
Chemical Shift (ppm)

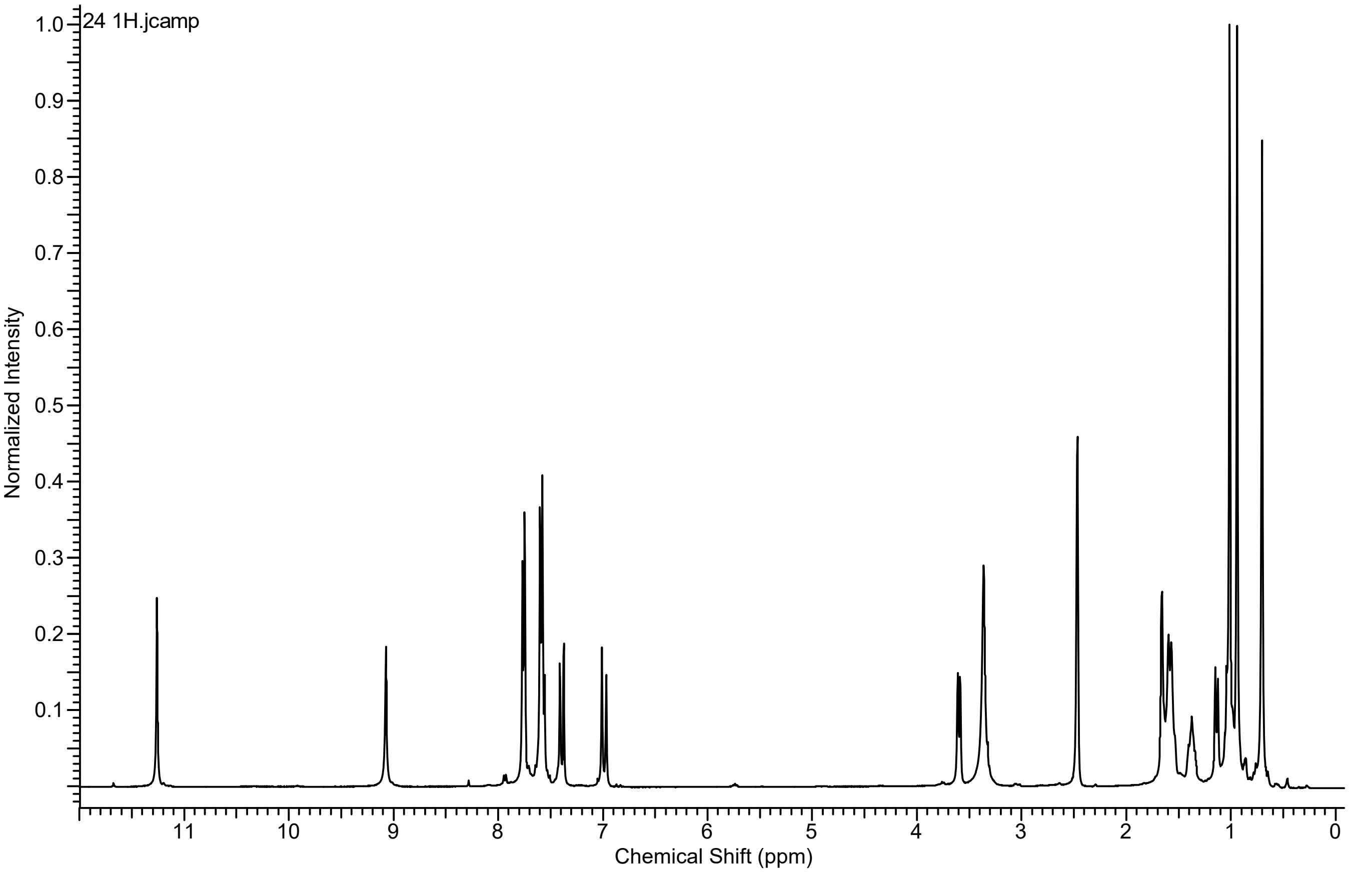
24 1H.jcamp
Normalized Intensity
Chemical Shift (ppm)

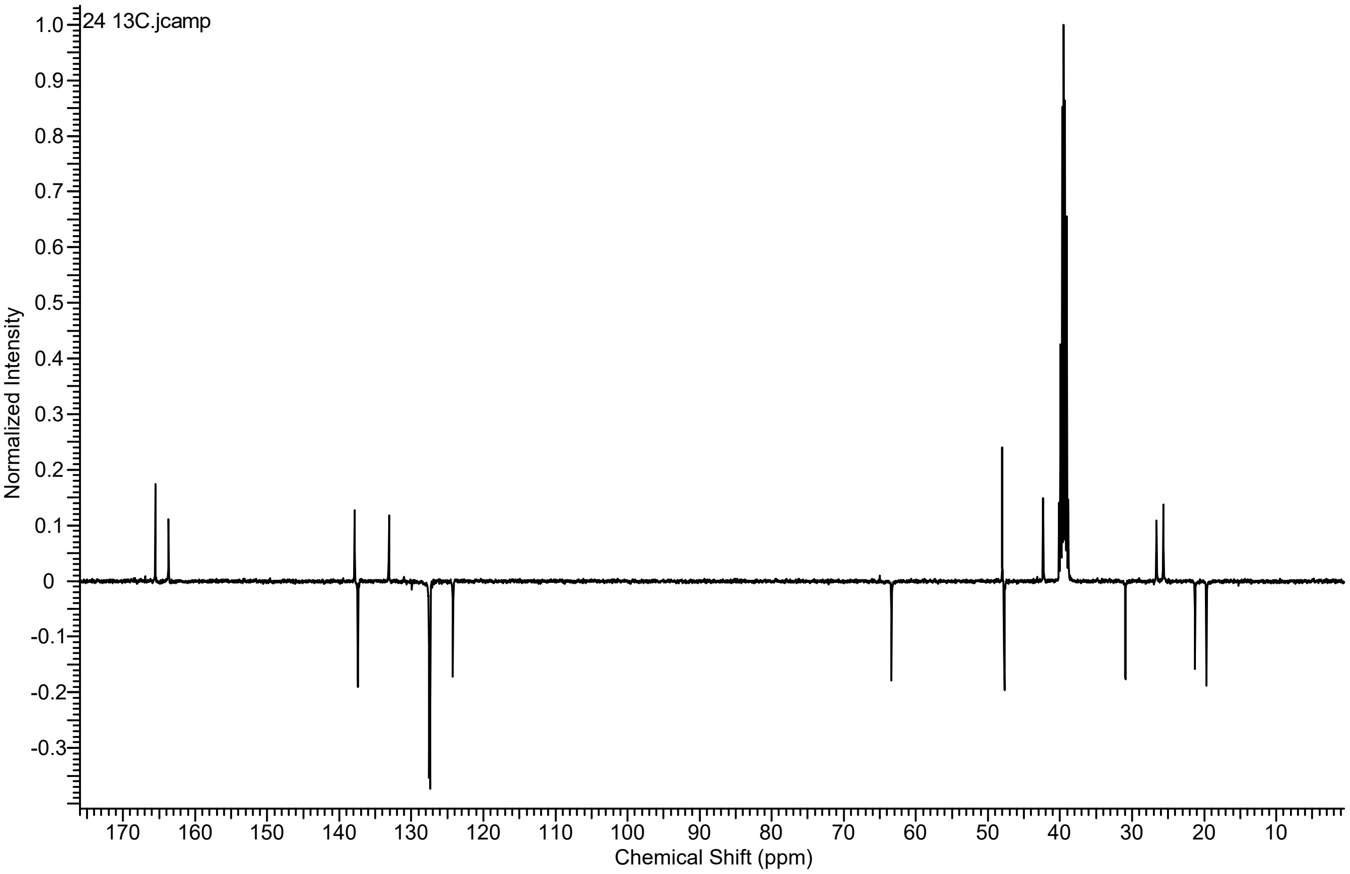
24 13C.jcamp
Normalized Intensity
Chemical Shift (ppm)

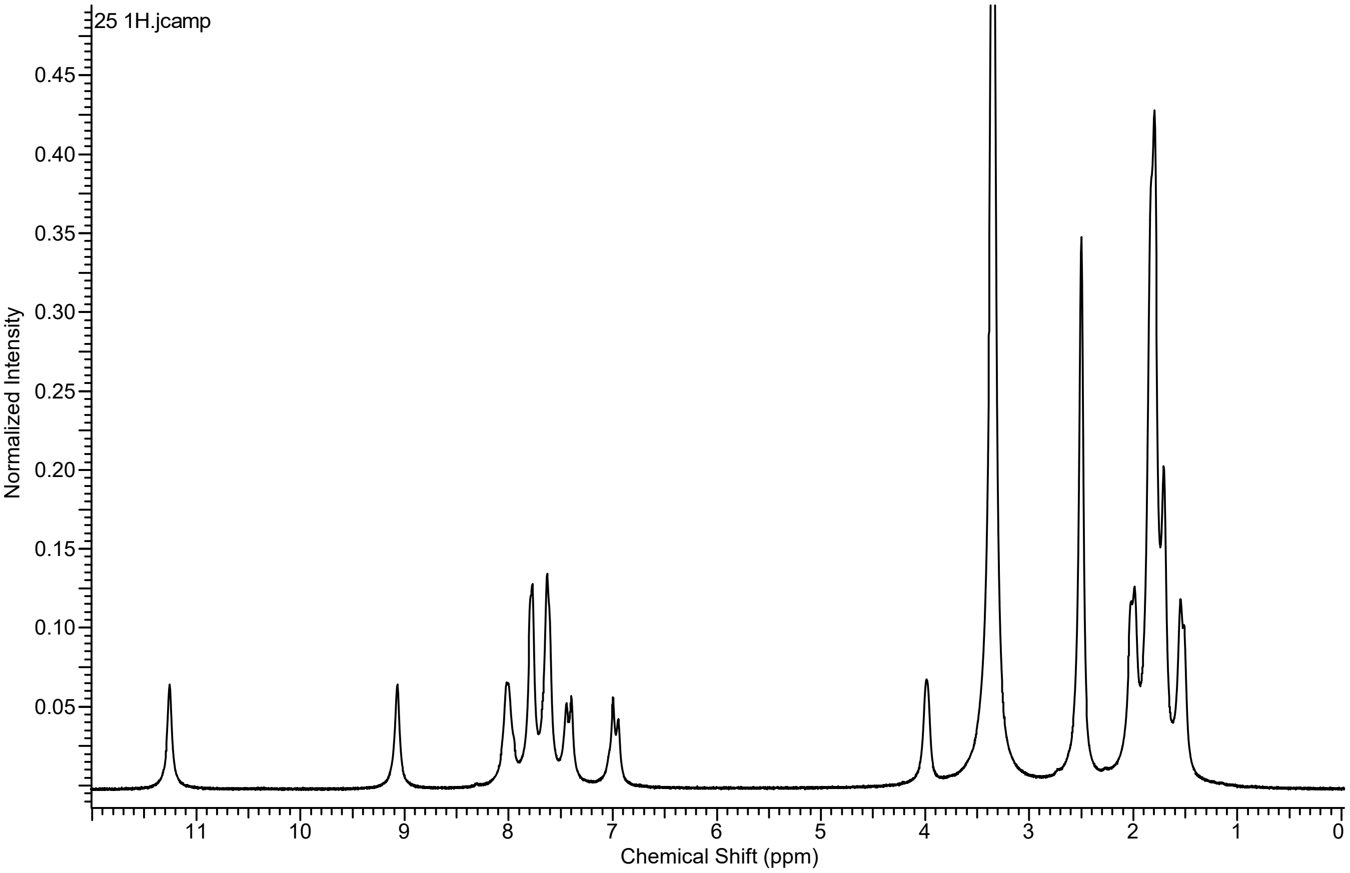
25 1H.jcamp
Normalized Intensity
Chemical Shift (ppm)

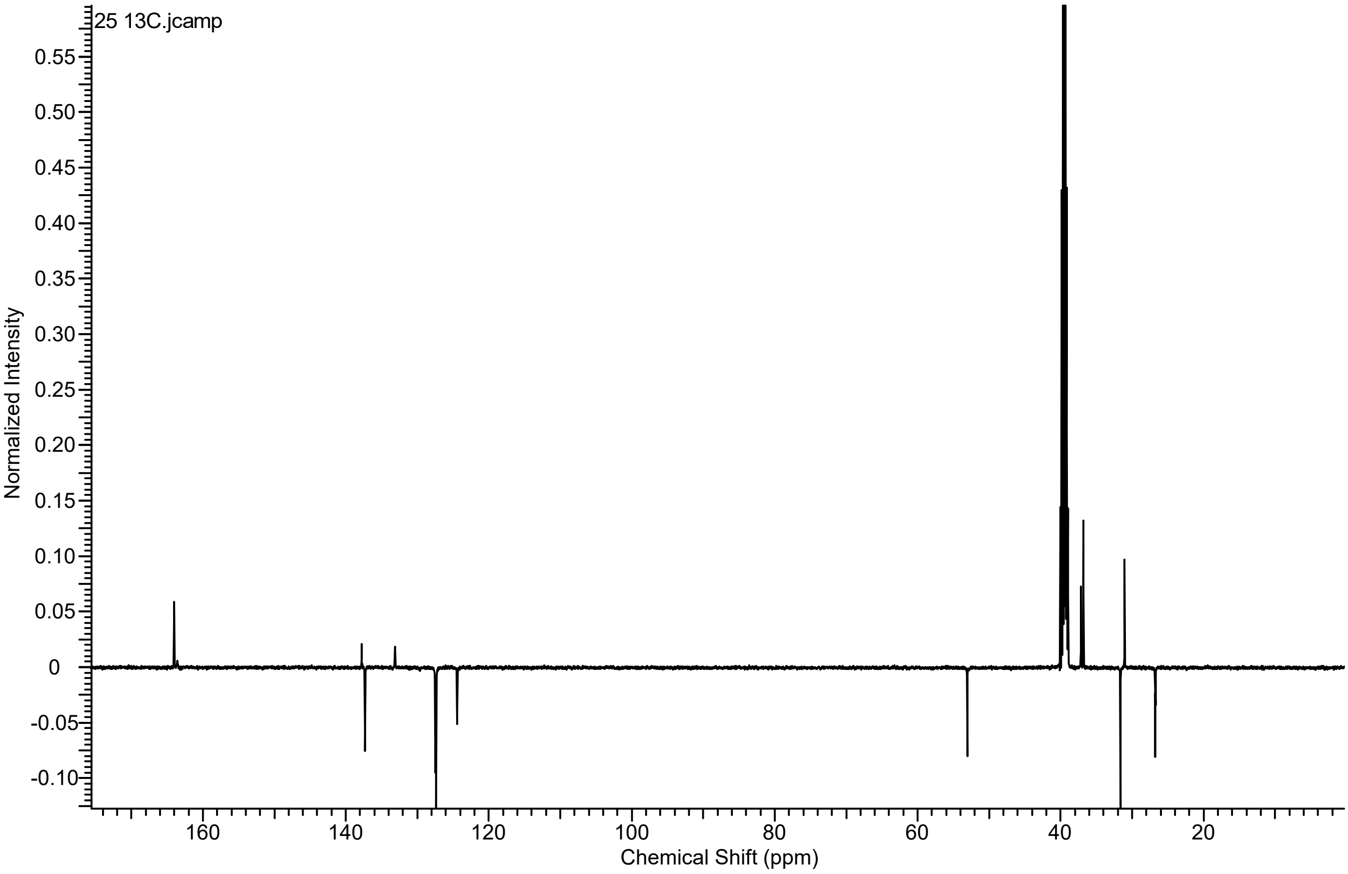

Figure S1. ^{1}H and ^{13}C NMR spectrums of compounds.
